# Bis(2,2′-bipyridine)(2-hy­droxy-2,2-diphenyl­acetato)­copper(II) nitrate dihydrate

**DOI:** 10.1107/S1600536810035555

**Published:** 2010-09-11

**Authors:** Xiao-Jun Wang, Chun Zheng, Shao-Wei Mai, Xuan Xu, Yi-Fan Luo

**Affiliations:** a School of Chemistry and Environment, South China Normal University, Guangzhou 510006, People’s Republic of China

## Abstract

In the title complex, [Cu(C_14_H_11_O_3_)(C_10_H_8_N_2_)_2_]NO_3_·2H_2_O, the Cu^II^ atom is coordinated by four N atoms from two 2,2′-bipyridine ligands and two O atoms from one benzilate ligand in a distorted octa­hedral geometry. A supra­molecular network is formed *via* inter­molecular O—H⋯O and C—H⋯O hydrogen-bonding inter­actions. π–π stacking inter­actions between neighboring pyridine rings are also present, the centroid—centroid distance being 3.808 (2) Å.

## Related literature

For related structures, see: Carballo *et al.* (2005 ▶); Herrmann *et al.* (1994 ▶); Qiu *et al.* (2007 ▶).
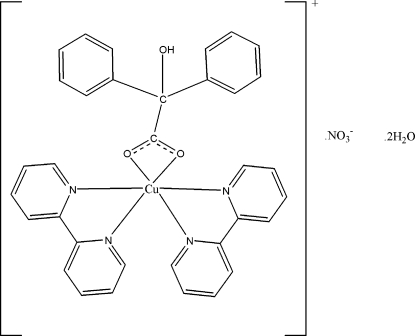


## Experimental

### 

#### Crystal data

[Cu(C_14_H_11_O_3_)(C_10_H_8_N_2_)_2_]NO_3_·2H_2_O
*M* *_r_* = 701.18
Monoclinic, 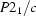

*a* = 10.612 (2) Å
*b* = 25.758 (6) Å
*c* = 12.322 (3) Å
β = 108.220 (3)°
*V* = 3199.3 (13) Å^3^
*Z* = 4
Mo *K*α radiation
μ = 0.74 mm^−1^
*T* = 296 K
0.23 × 0.21 × 0.19 mm

#### Data collection

Bruker APEXII area-detector diffractometer
Absorption correction: multi-scan (*SADABS*; Bruker, 2005 ▶) *T*_min_ = 0.848, *T*_max_ = 0.872
16165 measured reflections
5750 independent reflections
3659 reflections with *I* > 2σ(*I*)
*R*_int_ = 0.057

#### Refinement

*R*[*F* ^2^ > 2σ(*F* ^2^)] = 0.055
*wR*(*F* ^2^) = 0.138
*S* = 1.00
5750 reflections
434 parameters
6 restraints
H-atom parameters constrained
Δρ_max_ = 0.57 e Å^−3^
Δρ_min_ = −0.51 e Å^−3^

### 

Data collection: *APEX2* (Bruker, 2004 ▶); cell refinement: *SAINT* (Bruker, 2004 ▶); data reduction: *SAINT*; program(s) used to solve structure: *SHELXS97* (Sheldrick, 2008 ▶); program(s) used to refine structure: *SHELXL97* (Sheldrick, 2008 ▶); molecular graphics: *SHELXTL* (Sheldrick, 2008 ▶); software used to prepare material for publication: *SHELXTL*.

## Supplementary Material

Crystal structure: contains datablocks I, global. DOI: 10.1107/S1600536810035555/pv2323sup1.cif

Structure factors: contains datablocks I. DOI: 10.1107/S1600536810035555/pv2323Isup2.hkl

Additional supplementary materials:  crystallographic information; 3D view; checkCIF report

## Figures and Tables

**Table 1:** Hydrogen-bond geometry (Å, °)

*D*—H⋯*A*	*D*—H	H⋯*A*	*D*⋯*A*	*D*—H⋯*A*
O2*W*—H3*W*⋯O5^i^	0.85	2.16	2.844 (6)	138
O1*W*—H1*W*⋯O4^ii^	0.85	2.07	2.884 (7)	159
O2*W*—H3*W*⋯O1*W*^iii^	0.85	2.59	3.041 (7)	114
O1*W*—H2*W*⋯O2*W*^iii^	0.85	2.46	3.041 (7)	126
O1*W*—H2*W*⋯O4^iv^	0.85	2.28	2.856 (6)	125
O3—H3⋯O6^v^	0.82	2.48	3.210 (5)	149
O3—H3⋯O1	0.82	2.10	2.597 (4)	119
C10—H10⋯O1*W*	0.93	2.41	3.341 (7)	174
C8—H8⋯O3^iv^	0.93	2.54	3.389 (6)	152
C4—H4⋯O5^vi^	0.93	2.59	3.488 (6)	162
C12—H12⋯O5^vii^	0.93	2.38	3.285 (7)	165
C14—H14⋯O1^viii^	0.93	2.56	3.420 (6)	155
C17—H17⋯O1^viii^	0.93	2.39	3.270 (5)	159

Symmetry codes: (i) 
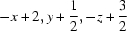
; (ii) 
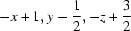
; (iii) 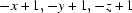; (iv) 
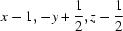
; (v) 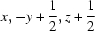; (vi) 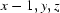; (vii) 
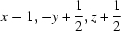
; (viii) 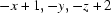.

**Fig. 1.**
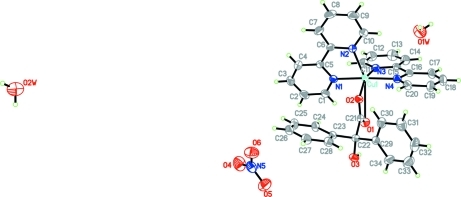
The molecular structure of the asymmetric unit of (I) showing the atomic-numbering scheme. Displacement ellipsoids are drawn at the 30% probability level.

**Fig. 2.**
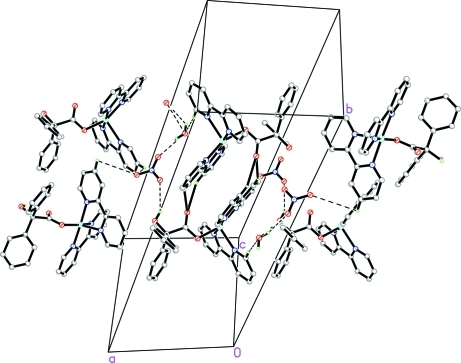
A view of the three-dimensional supramolecular network of the title compound, hydrogen bonds are shown as dashed linesand. The H-atoms not involved in H-bonds have been excluded for clarity.

## supplementary crystallographic information

### Comment

Hydrogen-bonding interactions between ligands are specific and directional. In this context, 2,2'-bipyridine and benzilic acid are excellent candidates for construction of three-demensional network motifs, and can simultaneously coordinate metal ions (Carballo *et al.*, 2005; Herrmann *et al.*, 1994; Qiu *et al.*, 2007). Herein, we report the crystal structure of a new coordination polymer, (I).

In (I), the Cu^II^ centre is coordinated by two oxygen atoms from one benzilate ligand and four N atoms from two 2,2'-bipyridine ligands (Fig. 1), and represents a distorted octahedral geometry. The Cu—N distances range from 1.982 (3) to 2.174 (3) Å, and the Cu—O distances are 1.982 (3) and 2.744 (3) Å, respectively. However, the O—Cu—N and N—Cu—N angles fall in the range from 89.06 (1) to 158.18 (1) ° and 77.87 (1) to 175.10 (1) °, respectively. Intermolecular O—H···O and C—H···O hydrogen bonding interactions (Table 1) link each asymmetric unit to form a three-dimensional supramolecular network motif (Fig. 2) in (0 0 1) plane, which is stabilized by π-π stacking interactions between neighboring pyridyl rings (the centriod—centriod distance is 3.808 Å).

### Experimental

A mixture of CuNO_3_ (0.063 g, 0.5mmol), 2,2'-bipyridine (0.078 g; 0.5 mmol), benzilic acid (0.114 g; 0.5 mmol), water (10 mL) was stirred vigorously for 60 min and the blue block crystals were obtained by evaporating mother liquor.

### Refinement

Water H atoms and hydroxyl H atoms were tentatively located from difference Fourier maps and were refined with distance restraints of O–H = 0.84 and 0.82 Å, respectively, H···H = 1.35 Å, and *U*_iso_(H) = 1.5 *U*_eq_(O). Carbon-bound H atoms were placed at calculated positions and were treated as riding on the parent C atoms with C—H = 0.93 Å, and with *U*_iso_(H) = 1.2 *U*_eq_(C).

### Crystal data

**Table:**

[Cu(C_14_H_11_O_3_)(C_10_H_8_N_2_)_2_]NO_3_·2H_2_O	*F*(000) = 1452
*M**_r_* = 701.18	*D*_x_ = 1.456 Mg m^−^^3^
Monoclinic, *P*2_1_/*c*	Mo *K*α radiation, λ = 0.71073 Å
Hall symbol: -P 2ybc	Cell parameters from 2592 reflections
*a* = 10.612 (2) Å	θ = 2.2–22.7°
*b* = 25.758 (6) Å	µ = 0.74 mm^−^^1^
*c* = 12.322 (3) Å	*T* = 296 K
β = 108.220 (3)°	Block, blue
*V* = 3199.3 (13) Å^3^	0.23 × 0.21 × 0.19 mm
*Z* = 4	

### Data collection

**Table:**

Bruker APEXII area-detector diffractometer	5750 independent reflections
Radiation source: fine-focus sealed tube	3659 reflections with *I* > 2σ(*I*)
graphite	*R*_int_ = 0.057
φ and ω scans	θ_max_ = 25.2°, θ_min_ = 1.6°
Absorption correction: multi-scan (*SADABS*; Bruker, 2005)	*h* = −12→9
*T*_min_ = 0.848, *T*_max_ = 0.872	*k* = −30→30
16165 measured reflections	*l* = −14→13

### Refinement

**Table:**

Refinement on *F*^2^	Primary atom site location: structure-invariant direct methods
Least-squares matrix: full	Secondary atom site location: difference Fourier map
*R*[*F*^2^ > 2σ(*F*^2^)] = 0.055	Hydrogen site location: inferred from neighbouring sites
*wR*(*F*^2^) = 0.138	H-atom parameters constrained
*S* = 1.00	*w* = 1/[σ^2^(*F*_o_^2^) + (0.053*P*)^2^ + 2.8562*P*] where *P* = (*F*_o_^2^ + 2*F*_c_^2^)/3
5750 reflections	(Δ/σ)_max_ < 0.001
434 parameters	Δρ_max_ = 0.56 e Å^−^^3^
6 restraints	Δρ_min_ = −0.51 e Å^−^^3^

### Special details

**Table:**

Geometry. All esds (except the esd in the dihedral angle between two l.s. planes) are estimated using the full covariance matrix. The cell esds are taken into account individually in the estimation of esds in distances, angles and torsion angles; correlations between esds in cell parameters are only used when they are defined by crystal symmetry. An approximate (isotropic) treatment of cell esds is used for estimating esds involving l.s. planes.
Refinement. Refinement of F^2^ against ALL reflections. The weighted R-factor wR and goodness of fit S are based on F^2^, conventional R-factors R are based on F, with F set to zero for negative F^2^. The threshold expression of F^2^ > 2sigma(F^2^) is used only for calculating R-factors(gt) etc. and is not relevant to the choice of reflections for refinement. R-factors based on F^2^ are statistically about twice as large as those based on F, and R- factors based on ALL data will be even larger.

### Fractional atomic coordinates and isotropic or equivalent isotropic displacement parameters (Å^2^)

**Table:**

	*x*	*y*	*z*	*U*_iso_*/*U*_eq_	
Cu1	0.41218 (5)	0.128914 (18)	0.88185 (4)	0.03907 (17)	
O1	0.6773 (3)	0.12292 (11)	1.0021 (2)	0.0493 (7)	
O2	0.5624 (3)	0.15418 (10)	0.8330 (2)	0.0416 (7)	
O3	0.9044 (3)	0.15772 (12)	0.9953 (2)	0.0487 (7)	
H3	0.8800	0.1396	1.0397	0.073*	
O4	0.9802 (5)	0.43639 (17)	0.8832 (4)	0.1032 (14)	
O5	1.1068 (4)	0.38476 (16)	0.8257 (4)	0.0910 (13)	
O6	0.9367 (4)	0.42004 (17)	0.7058 (4)	0.0890 (12)	
O1W	0.1694 (5)	0.02577 (18)	0.5874 (4)	0.1147 (15)	
H2W	0.1658	0.0353	0.5204	0.172*	
H1W	0.1207	−0.0011	0.5773	0.172*	
O2W	0.6493 (5)	0.92807 (19)	0.5360 (4)	0.1224 (16)	
H4W	0.6297	0.9527	0.4877	0.184*	
H3W	0.7332	0.9259	0.5527	0.184*	
N1	0.4082 (3)	0.19785 (13)	0.9567 (3)	0.0407 (8)	
N2	0.2541 (3)	0.16914 (13)	0.7536 (3)	0.0422 (8)	
N3	0.3174 (3)	0.08879 (13)	0.9731 (3)	0.0432 (8)	
N4	0.4228 (3)	0.05833 (12)	0.8213 (3)	0.0414 (8)	
N5	1.0072 (5)	0.41464 (17)	0.8048 (4)	0.0656 (11)	
C1	0.4840 (4)	0.20910 (18)	1.0623 (4)	0.0509 (11)	
H1	0.5372	0.1831	1.1055	0.061*	
C2	0.4873 (5)	0.2571 (2)	1.1103 (4)	0.0637 (14)	
H2	0.5397	0.2634	1.1851	0.076*	
C3	0.4119 (5)	0.2954 (2)	1.0458 (5)	0.0705 (16)	
H3A	0.4146	0.3287	1.0753	0.085*	
C4	0.3318 (5)	0.28473 (18)	0.9368 (5)	0.0644 (14)	
H4	0.2789	0.3107	0.8928	0.077*	
C5	0.3301 (4)	0.23523 (16)	0.8930 (4)	0.0442 (10)	
C6	0.2447 (4)	0.21925 (16)	0.7790 (3)	0.0415 (10)	
C7	0.1595 (5)	0.25249 (19)	0.7035 (4)	0.0591 (13)	
H7	0.1552	0.2873	0.7221	0.071*	
C8	0.0810 (5)	0.2338 (2)	0.6007 (4)	0.0714 (15)	
H8	0.0226	0.2557	0.5488	0.086*	
C9	0.0892 (5)	0.1829 (2)	0.5753 (4)	0.0701 (15)	
H9	0.0365	0.1695	0.5059	0.084*	
C10	0.1764 (5)	0.15173 (18)	0.6536 (4)	0.0547 (12)	
H10	0.1814	0.1168	0.6360	0.066*	
C11	0.2639 (5)	0.10780 (19)	1.0494 (4)	0.0549 (12)	
H11	0.2707	0.1433	1.0639	0.066*	
C12	0.1999 (5)	0.0782 (2)	1.1073 (4)	0.0670 (14)	
H12	0.1633	0.0928	1.1596	0.080*	
C13	0.1914 (6)	0.0260 (2)	1.0854 (5)	0.0752 (16)	
H13	0.1497	0.0044	1.1242	0.090*	
C14	0.2437 (5)	0.0054 (2)	1.0070 (4)	0.0623 (13)	
H14	0.2363	−0.0300	0.9911	0.075*	
C15	0.3082 (4)	0.03765 (16)	0.9512 (4)	0.0434 (10)	
C16	0.3693 (4)	0.02037 (16)	0.8674 (3)	0.0417 (10)	
C17	0.3744 (5)	−0.03074 (17)	0.8344 (4)	0.0560 (12)	
H17	0.3370	−0.0569	0.8664	0.067*	
C18	0.4351 (5)	−0.04255 (18)	0.7543 (4)	0.0639 (14)	
H18	0.4390	−0.0768	0.7316	0.077*	
C19	0.4897 (5)	−0.00371 (18)	0.7080 (4)	0.0626 (13)	
H19	0.5320	−0.0109	0.6541	0.075*	
C20	0.4803 (5)	0.04618 (17)	0.7434 (4)	0.0518 (12)	
H20	0.5161	0.0728	0.7112	0.062*	
C21	0.6700 (4)	0.14445 (14)	0.9122 (4)	0.0387 (10)	
C22	0.8000 (4)	0.16241 (15)	0.8909 (3)	0.0376 (9)	
C23	0.7908 (4)	0.21966 (15)	0.8583 (3)	0.0393 (10)	
C24	0.7320 (5)	0.25356 (16)	0.9140 (4)	0.0506 (11)	
H24	0.6962	0.2409	0.9687	0.061*	
C25	0.7252 (5)	0.30584 (18)	0.8902 (5)	0.0645 (14)	
H25	0.6849	0.3282	0.9286	0.077*	
C26	0.7776 (5)	0.32494 (18)	0.8103 (4)	0.0606 (13)	
H26	0.7723	0.3602	0.7932	0.073*	
C27	0.8376 (5)	0.29183 (19)	0.7558 (4)	0.0610 (13)	
H27	0.8742	0.3048	0.7020	0.073*	
C28	0.8451 (4)	0.23917 (17)	0.7792 (4)	0.0511 (11)	
H28	0.8868	0.2171	0.7415	0.061*	
C29	0.8237 (4)	0.12686 (15)	0.7997 (3)	0.0392 (9)	
C30	0.7340 (5)	0.12378 (17)	0.6913 (4)	0.0546 (12)	
H30	0.6588	0.1447	0.6715	0.066*	
C31	0.7548 (6)	0.0900 (2)	0.6122 (4)	0.0709 (15)	
H31	0.6929	0.0877	0.5397	0.085*	
C32	0.8672 (7)	0.0595 (2)	0.6401 (6)	0.0791 (17)	
H32	0.8820	0.0370	0.5864	0.095*	
C33	0.9555 (6)	0.0627 (2)	0.7457 (6)	0.0794 (17)	
H33	1.0314	0.0422	0.7646	0.095*	
C34	0.9351 (5)	0.09591 (18)	0.8255 (4)	0.0592 (13)	
H34	0.9971	0.0975	0.8980	0.071*	

### Atomic displacement parameters (Å^2^)

**Table:**

	*U*^11^	*U*^22^	*U*^33^	*U*^12^	*U*^13^	*U*^23^
Cu1	0.0364 (3)	0.0342 (3)	0.0450 (3)	−0.0020 (2)	0.0105 (2)	0.0002 (2)
O1	0.0513 (19)	0.0515 (18)	0.0466 (18)	0.0013 (15)	0.0173 (15)	0.0154 (14)
O2	0.0374 (17)	0.0392 (16)	0.0461 (17)	−0.0033 (13)	0.0101 (14)	0.0019 (13)
O3	0.0391 (17)	0.056 (2)	0.0426 (17)	−0.0052 (14)	0.0012 (14)	0.0060 (14)
O4	0.122 (4)	0.099 (3)	0.101 (3)	−0.014 (3)	0.052 (3)	−0.037 (3)
O5	0.083 (3)	0.087 (3)	0.106 (3)	0.021 (2)	0.035 (3)	0.023 (2)
O6	0.072 (3)	0.113 (3)	0.075 (3)	−0.002 (2)	0.013 (2)	0.013 (2)
O1W	0.105 (4)	0.113 (4)	0.120 (4)	−0.008 (3)	0.025 (3)	−0.028 (3)
O2W	0.091 (3)	0.142 (4)	0.126 (4)	0.007 (3)	0.022 (3)	0.002 (3)
N1	0.0338 (19)	0.045 (2)	0.043 (2)	−0.0035 (16)	0.0108 (17)	−0.0041 (16)
N2	0.036 (2)	0.040 (2)	0.046 (2)	0.0015 (16)	0.0063 (17)	−0.0025 (16)
N3	0.037 (2)	0.043 (2)	0.049 (2)	0.0013 (16)	0.0127 (17)	0.0030 (16)
N4	0.040 (2)	0.040 (2)	0.043 (2)	−0.0030 (16)	0.0110 (17)	0.0023 (16)
N5	0.069 (3)	0.060 (3)	0.072 (3)	−0.016 (2)	0.028 (3)	0.000 (2)
C1	0.042 (3)	0.057 (3)	0.051 (3)	−0.003 (2)	0.010 (2)	−0.008 (2)
C2	0.050 (3)	0.075 (4)	0.066 (3)	−0.008 (3)	0.017 (3)	−0.027 (3)
C3	0.061 (3)	0.058 (3)	0.091 (4)	−0.004 (3)	0.021 (3)	−0.039 (3)
C4	0.058 (3)	0.047 (3)	0.084 (4)	0.008 (2)	0.016 (3)	−0.007 (3)
C5	0.035 (2)	0.042 (2)	0.055 (3)	0.001 (2)	0.014 (2)	−0.006 (2)
C6	0.034 (2)	0.046 (3)	0.044 (3)	0.0041 (19)	0.011 (2)	0.000 (2)
C7	0.051 (3)	0.056 (3)	0.065 (3)	0.016 (2)	0.011 (3)	0.012 (2)
C8	0.054 (3)	0.087 (4)	0.060 (4)	0.023 (3)	−0.002 (3)	0.014 (3)
C9	0.053 (3)	0.090 (4)	0.051 (3)	0.006 (3)	−0.007 (3)	−0.005 (3)
C10	0.050 (3)	0.051 (3)	0.054 (3)	0.001 (2)	0.003 (2)	−0.006 (2)
C11	0.050 (3)	0.059 (3)	0.061 (3)	0.000 (2)	0.026 (3)	0.000 (2)
C12	0.061 (3)	0.081 (4)	0.069 (3)	−0.001 (3)	0.034 (3)	0.005 (3)
C13	0.083 (4)	0.078 (4)	0.076 (4)	−0.013 (3)	0.041 (3)	0.018 (3)
C14	0.067 (3)	0.056 (3)	0.066 (3)	−0.006 (3)	0.023 (3)	0.011 (2)
C15	0.035 (2)	0.043 (3)	0.046 (3)	−0.0045 (19)	0.003 (2)	0.009 (2)
C16	0.036 (2)	0.040 (2)	0.043 (3)	−0.0039 (18)	0.003 (2)	0.0070 (19)
C17	0.064 (3)	0.037 (3)	0.065 (3)	−0.009 (2)	0.018 (3)	0.004 (2)
C18	0.075 (4)	0.040 (3)	0.072 (4)	−0.001 (3)	0.018 (3)	−0.007 (2)
C19	0.071 (4)	0.049 (3)	0.073 (3)	0.000 (3)	0.030 (3)	−0.010 (2)
C20	0.054 (3)	0.049 (3)	0.054 (3)	−0.009 (2)	0.018 (2)	−0.003 (2)
C21	0.041 (3)	0.030 (2)	0.044 (3)	0.0003 (18)	0.012 (2)	−0.0027 (18)
C22	0.033 (2)	0.041 (2)	0.037 (2)	−0.0013 (18)	0.0094 (19)	0.0019 (18)
C23	0.035 (2)	0.035 (2)	0.044 (2)	−0.0055 (18)	0.008 (2)	−0.0010 (18)
C24	0.060 (3)	0.040 (3)	0.057 (3)	−0.007 (2)	0.025 (2)	−0.003 (2)
C25	0.070 (4)	0.045 (3)	0.080 (4)	−0.001 (3)	0.025 (3)	−0.008 (3)
C26	0.055 (3)	0.041 (3)	0.075 (4)	−0.006 (2)	0.006 (3)	0.007 (3)
C27	0.061 (3)	0.053 (3)	0.067 (3)	−0.013 (3)	0.018 (3)	0.017 (3)
C28	0.051 (3)	0.050 (3)	0.056 (3)	−0.005 (2)	0.022 (2)	0.003 (2)
C29	0.039 (2)	0.036 (2)	0.047 (2)	−0.0025 (19)	0.019 (2)	0.0019 (19)
C30	0.067 (3)	0.050 (3)	0.047 (3)	0.003 (2)	0.018 (2)	−0.003 (2)
C31	0.095 (5)	0.065 (3)	0.054 (3)	−0.010 (3)	0.025 (3)	−0.009 (3)
C32	0.107 (5)	0.058 (3)	0.091 (5)	0.005 (3)	0.058 (4)	−0.012 (3)
C33	0.073 (4)	0.074 (4)	0.099 (5)	0.016 (3)	0.040 (4)	−0.001 (3)
C34	0.048 (3)	0.063 (3)	0.069 (3)	0.010 (2)	0.022 (3)	0.002 (3)

### Geometric parameters (Å, °)

**Table:**

Cu1—O2	1.982 (3)	C11—H11	0.9300
Cu1—N4	1.982 (3)	C12—C13	1.369 (7)
Cu1—N1	2.007 (3)	C12—H12	0.9300
Cu1—N3	2.013 (3)	C13—C14	1.362 (7)
Cu1—N2	2.174 (3)	C13—H13	0.9300
O1—C21	1.220 (5)	C14—C15	1.388 (6)
O2—C21	1.274 (5)	C14—H14	0.9300
O3—C22	1.417 (4)	C15—C16	1.450 (6)
O3—H3	0.8200	C16—C17	1.384 (6)
O4—N5	1.225 (5)	C17—C18	1.370 (6)
O5—N5	1.267 (5)	C17—H17	0.9300
O6—N5	1.223 (5)	C18—C19	1.367 (7)
O1W—H2W	0.8499	C18—H18	0.9300
O1W—H1W	0.8501	C19—C20	1.371 (6)
O2W—H4W	0.8499	C19—H19	0.9300
O2W—H3W	0.8499	C20—H20	0.9300
N1—C1	1.330 (5)	C21—C22	1.553 (6)
N1—C5	1.350 (5)	C22—C23	1.523 (5)
N2—C10	1.328 (5)	C22—C29	1.530 (5)
N2—C6	1.339 (5)	C23—C28	1.374 (6)
N3—C11	1.334 (5)	C23—C24	1.375 (6)
N3—C15	1.342 (5)	C24—C25	1.375 (6)
N4—C20	1.326 (5)	C24—H24	0.9300
N4—C16	1.343 (5)	C25—C26	1.365 (7)
C1—C2	1.366 (6)	C25—H25	0.9300
C1—H1	0.9300	C26—C27	1.360 (7)
C2—C3	1.359 (7)	C26—H26	0.9300
C2—H2	0.9300	C27—C28	1.384 (6)
C3—C4	1.374 (7)	C27—H27	0.9300
C3—H3A	0.9300	C28—H28	0.9300
C4—C5	1.383 (6)	C29—C34	1.378 (6)
C4—H4	0.9300	C29—C30	1.379 (6)
C5—C6	1.473 (6)	C30—C31	1.375 (6)
C6—C7	1.375 (6)	C30—H30	0.9300
C7—C8	1.368 (7)	C31—C32	1.379 (8)
C7—H7	0.9300	C31—H31	0.9300
C8—C9	1.356 (7)	C32—C33	1.347 (8)
C8—H8	0.9300	C32—H32	0.9300
C9—C10	1.369 (6)	C33—C34	1.371 (7)
C9—H9	0.9300	C33—H33	0.9300
C10—H10	0.9300	C34—H34	0.9300
C11—C12	1.362 (6)		
			
O2—Cu1—N4	92.34 (13)	C12—C13—H13	119.8
O2—Cu1—N1	89.06 (12)	C13—C14—C15	119.5 (5)
N4—Cu1—N1	175.10 (14)	C13—C14—H14	120.3
O2—Cu1—N3	158.18 (12)	C15—C14—H14	120.3
N4—Cu1—N3	80.61 (14)	N3—C15—C14	120.2 (4)
N1—Cu1—N3	96.40 (14)	N3—C15—C16	115.1 (4)
O2—Cu1—N2	97.69 (12)	C14—C15—C16	124.7 (4)
N4—Cu1—N2	106.57 (13)	N4—C16—C17	120.5 (4)
N1—Cu1—N2	77.87 (13)	N4—C16—C15	114.9 (4)
N3—Cu1—N2	104.11 (13)	C17—C16—C15	124.7 (4)
C21—O2—Cu1	108.4 (3)	C18—C17—C16	119.6 (4)
C22—O3—H3	109.5	C18—C17—H17	120.2
H2W—O1W—H1W	104.6	C16—C17—H17	120.2
H4W—O2W—H3W	103.2	C19—C18—C17	119.6 (4)
C1—N1—C5	119.1 (4)	C19—C18—H18	120.2
C1—N1—Cu1	123.3 (3)	C17—C18—H18	120.2
C5—N1—Cu1	117.5 (3)	C18—C19—C20	118.1 (5)
C10—N2—C6	118.1 (4)	C18—C19—H19	120.9
C10—N2—Cu1	128.9 (3)	C20—C19—H19	120.9
C6—N2—Cu1	112.8 (3)	N4—C20—C19	123.2 (4)
C11—N3—C15	118.8 (4)	N4—C20—H20	118.4
C11—N3—Cu1	127.0 (3)	C19—C20—H20	118.4
C15—N3—Cu1	114.1 (3)	O1—C21—O2	125.0 (4)
C20—N4—C16	119.1 (4)	O1—C21—C22	118.8 (4)
C20—N4—Cu1	125.6 (3)	O2—C21—C22	116.3 (3)
C16—N4—Cu1	115.3 (3)	O3—C22—C23	106.8 (3)
O6—N5—O4	120.8 (5)	O3—C22—C29	110.7 (3)
O6—N5—O5	118.8 (5)	C23—C22—C29	113.4 (3)
O4—N5—O5	120.3 (5)	O3—C22—C21	107.8 (3)
N1—C1—C2	123.2 (5)	C23—C22—C21	110.2 (3)
N1—C1—H1	118.4	C29—C22—C21	107.7 (3)
C2—C1—H1	118.4	C28—C23—C24	118.6 (4)
C3—C2—C1	118.2 (5)	C28—C23—C22	122.3 (4)
C3—C2—H2	120.9	C24—C23—C22	119.0 (4)
C1—C2—H2	120.9	C23—C24—C25	121.2 (4)
C2—C3—C4	119.8 (5)	C23—C24—H24	119.4
C2—C3—H3A	120.1	C25—C24—H24	119.4
C4—C3—H3A	120.1	C26—C25—C24	120.0 (5)
C3—C4—C5	119.6 (5)	C26—C25—H25	120.0
C3—C4—H4	120.2	C24—C25—H25	120.0
C5—C4—H4	120.2	C27—C26—C25	119.3 (5)
N1—C5—C4	120.0 (4)	C27—C26—H26	120.4
N1—C5—C6	116.0 (4)	C25—C26—H26	120.4
C4—C5—C6	124.0 (4)	C26—C27—C28	121.2 (5)
N2—C6—C7	121.6 (4)	C26—C27—H27	119.4
N2—C6—C5	115.0 (3)	C28—C27—H27	119.4
C7—C6—C5	123.4 (4)	C23—C28—C27	119.8 (4)
C8—C7—C6	119.2 (5)	C23—C28—H28	120.1
C8—C7—H7	120.4	C27—C28—H28	120.1
C6—C7—H7	120.4	C34—C29—C30	118.2 (4)
C9—C8—C7	119.3 (5)	C34—C29—C22	120.2 (4)
C9—C8—H8	120.3	C30—C29—C22	121.6 (4)
C7—C8—H8	120.3	C31—C30—C29	120.5 (5)
C8—C9—C10	118.8 (5)	C31—C30—H30	119.7
C8—C9—H9	120.6	C29—C30—H30	119.7
C10—C9—H9	120.6	C30—C31—C32	120.1 (5)
N2—C10—C9	122.9 (4)	C30—C31—H31	119.9
N2—C10—H10	118.6	C32—C31—H31	119.9
C9—C10—H10	118.6	C33—C32—C31	119.5 (5)
N3—C11—C12	123.7 (5)	C33—C32—H32	120.3
N3—C11—H11	118.2	C31—C32—H32	120.3
C12—C11—H11	118.2	C32—C33—C34	120.9 (5)
C11—C12—C13	117.4 (5)	C32—C33—H33	119.6
C11—C12—H12	121.3	C34—C33—H33	119.6
C13—C12—H12	121.3	C33—C34—C29	120.8 (5)
C14—C13—C12	120.4 (5)	C33—C34—H34	119.6
C14—C13—H13	119.8	C29—C34—H34	119.6

### Hydrogen-bond geometry (Å, °)

**Table:**

*D*—H···*A*	*D*—H	H···*A*	*D*···*A*	*D*—H···*A*
O2W—H3W···O5^i^	0.85	2.16	2.844 (6)	138
O1W—H1W···O4^ii^	0.85	2.07	2.884 (7)	159
O2W—H3W···O1W^iii^	0.85	2.59	3.041 (7)	114
O1W—H2W···O2W^iii^	0.85	2.46	3.041 (7)	126
O1W—H2W···O4^iv^	0.85	2.28	2.856 (6)	125
O3—H3···O6^v^	0.82	2.48	3.210 (5)	149
O3—H3···O1	0.82	2.10	2.597 (4)	119
C20—H20···O2	0.93	2.53	3.019 (5)	113
C30—H30···O2	0.93	2.52	2.994 (5)	112
C34—H34···O3	0.93	2.35	2.728 (6)	104
C10—H10···O1W	0.93	2.41	3.341 (7)	174
C8—H8···O3^iv^	0.93	2.54	3.389 (6)	152
C4—H4···O5^vi^	0.93	2.59	3.488 (6)	162
C12—H12···O5^vii^	0.93	2.38	3.285 (7)	165
C14—H14···O1^viii^	0.93	2.56	3.420 (6)	155
C17—H17···O1^viii^	0.93	2.39	3.270 (5)	159

Symmetry codes: (i) −*x*+2, *y*+1/2, −*z*+3/2; (ii) −*x*+1, *y*−1/2, −*z*+3/2; (iii) −*x*+1, −*y*+1, −*z*+1; (iv) *x*−1, −*y*+1/2, *z*−1/2; (v) *x*, −*y*+1/2, *z*+1/2; (vi) *x*−1, *y*, *z*; (vii) *x*−1, −*y*+1/2, *z*+1/2; (viii) −*x*+1, −*y*, −*z*+2.
